# Downregulation of PA28α induces proteasome remodeling and results in resistance to proteasome inhibitors in multiple myeloma

**DOI:** 10.1038/s41408-020-00393-0

**Published:** 2020-12-14

**Authors:** Yanyan Gu, Benjamin G. Barwick, Mala Shanmugam, Craig C. Hofmeister, Jonathan Kaufman, Ajay Nooka, Vikas Gupta, Madhav Dhodapkar, Lawrence H. Boise, Sagar Lonial

**Affiliations:** 1 Department of Hematology and Medical Oncology, Emory University School of Medicine, 1365 Clifton Road, Atlanta, GA 30322 USA; 2 Winship Cancer Institute, Emory University, 1365 Clifton Road, Atlanta, GA 30322 USA

**Keywords:** Myeloma, Translational research

## Abstract

Protein homeostasis is critical for maintaining eukaryotic cell function as well as responses to intrinsic and extrinsic stress. The proteasome is a major portion of the proteolytic machinery in mammalian cells and plays an important role in protein homeostasis. Multiple myeloma (MM) is a plasma cell malignancy with high production of immunoglobulins and is especially sensitive to treatments that impact protein catabolism. Therapeutic agents such as proteasome inhibitors have demonstrated significant benefit for myeloma patients in all treatment phases. Here, we demonstrate that the 11S proteasome activator PA28α is upregulated in MM cells and is key for myeloma cell growth and proliferation. PA28α also regulates MM cell sensitivity to proteasome inhibitors. Downregulation of PA28α inhibits both proteasomal load and activity, resulting in a change in protein homeostasis less dependent on the proteasome and leads to cell resistance to proteasome inhibitors. Thus, our findings suggest an important role of PA28α in MM biology, and also provides a new approach for targeting the ubiquitin-proteasome system and ultimately sensitivity to proteasome inhibitors.

## Introduction

Multiple myeloma (MM) is the second most common hematologic malignancy in the United States. Active treatments include CD38 antibodies, immunomodulatory imide drugs, dexamethasone, and proteasome inhibitors (PI), which have improved patient outcomes. Bortezomib, carfilzomib, and the oral proteasome inhibitor ixazomib are approved for the treatment of MM patients each with different formulations and adverse events^1–3^. Despite the clinical benefits of PIs, most patients inevitably develop resistance and relapse, and the underlying mechanisms of drug resistance remain unclear^4–8^.

Initial sensitivity to PIs is a consequence of aberrant protein homeostasis in MM cells. As a consequence of their high rate of immunoglobulin synthesis, myeloma cells are dependent upon protein folding, trafficking, and degradation systems to maintain survival. Perturbation of protein homeostasis can ultimately induce myeloma cell death^9,10^. The ubiquitin–proteasome system (UPS) is a central mechanism for protein homeostasis^11^ and its pivotal role in protein catabolism renders it an attractive target in myeloma therapy.

A functional proteasome is composed of a hollow cylindrical 20S proteasome (core particle, CP) with a regulatory particle (RP) on one or both ends^12^. Three different proteasome regulators can activate a 20S proteasome, namely, PA700 (19S), REG/PA28 family (α, β, γ), and PA200^13–15^. PA28γ (REGγ, 11Sγ, or PSME3) is localized in the nucleus and forms homo-heptameric rings. PA28γ is not IFN-γ inducible but participates in chromosomal stability maintenance^16^, as well as ATP and ubiquitin-independent degradation of some molecules, such as SRC-3 and P21^17,18^. PA28α (REGα, 11Sα, or PSME1) and PA28β (REGβ, 11Sβ, or PSME2) form hetero-heptameric rings binding to one or two ends of the 20S proteasome to form 11S/20S/11S or hybrid proteasome. PA28α and β are IFN-γ inducible and function as part of MHC class I antigen presentation^19–21^. PA28α and β are basally expressed in all tissues, but at higher levels in antigen-presenting cells. Our analysis of plasma cells from MM patients with healthy donors has demonstrated increased expression of the 11S proteasome in malignant plasma cells. Although PA28α and β form a heptameric complex to activate proteasome activity within cells, only recombinant PA28α can directly stimulate proteasome peptidase activity. PA28β does not exert a stimulatory effect but enhances the affinity of 11S with 20S proteasome^22–25^. Our recent finding has demonstrated that perturbation of 11S/20S interaction can regulate proteasome function and sensitivity to PIs in MM^8^. Given the importance of the 11S proteasome in malignant plasma cells, we sought to identify the function of the 11S subunits, especially the α subunit, in myeloma biology. We generated PA28α stable knockdown myeloma cells and have demonstrated that PA28α is important for myeloma cell proliferation and sensitivity to PIs. Silencing of PA28α suppresses MM cell proliferation and induces PI resistance. The mechanism of PI resistance is distinct from which has been reported with knocking down 20S or 19S proteasome subunits. Knockdown of PA28α inhibited proteasome activity, but also decreased proteasome load, resulting in a change in protein homeostasis which is less dependent on the proteasome. Together, these data demonstrate that the 11S proteasome is an important target in myeloma cell biology and provides a new opportunity to enhance the efficacy of currently available treatment methods.

## Results

### PA28α upregulation is a hallmark of human MM

**Fig. 1:**
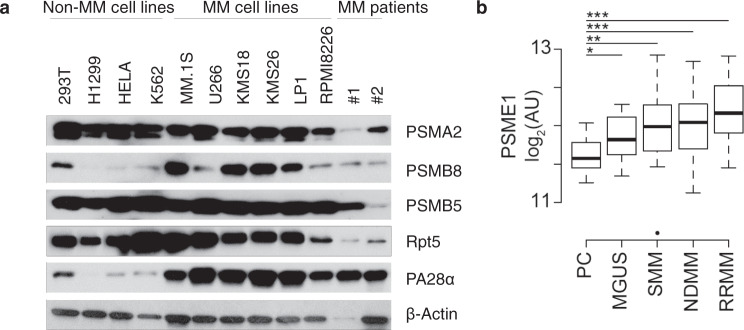
PA28α is upregulated in human multiple myeloma cells. **a** Western blot of proteasome subunits expression on cell lysate from a variety of cell lines and primary MM cells, β-actin as a loading control. **b** Boxplot of PSME1 expression data in normal plasma cells (PC, *n* = 15), monoclonal gammopathy of undetermined significance (MGUS, *n* = 22), smoldering multiple myeloma (SMM, *n* = 24), newly diagnosed multiple myeloma (NDMM, *n* = 73), and relapsed/refractory multiple myeloma (RRMM, *n* = 28) from Chng et al.^25^. *P < 0.05, **P < 0.01, ***P < 0.001, determined using linear regression with disease stage as a factor.

We first evaluated proteasome subunit expression levels in a group of human myeloma cell lines, primary cells from two MM patients, and a few non-MM cell lines. Among all tested cells, the 20S proteasome subunit, PSMA2, PSMB5, and the 19S proteasome subunit Rpt5 were constitutively expressed, whereas the 11S subunit PA28α was specifically expressed at high levels only in MM cells (Fig.1a). We next evaluated the expression of *PSME1* (which encodes PA28α) in plasma cells from healthy donors, monoclonal gammopathy of undetermined significance (MGUS), smoldering multiple myeloma (SMM), newly diagnosed multiple myeloma (NDMM), and relapsed/refractory multiple myeloma (RRMM) using data from Chng et al.^26^. This demonstrated that *PSME1* was progressively upregulated with disease progression (Fig.1b), suggesting that PA28α is associated with MM cell transformation and tumor progression.

### PA28α regulates multiple myeloma sensitivity to PI

**Fig. 2:**
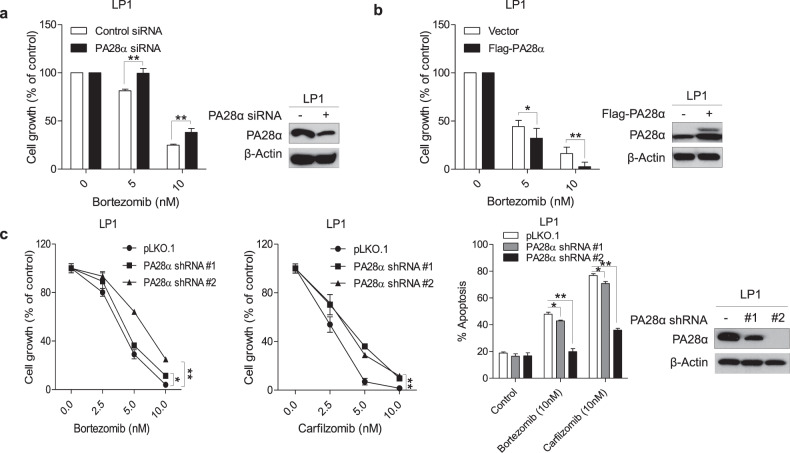
PA28α regulates multiple myeloma sensitivity to PIs. **a** LP1 cells were transfected with PA28α siRNA for 72 h, cells were then treated with bortezomib for 24 h and MTT assay was performed to analyze cell growth. **b** LP1 cells were transfected with Flag-PA28α for 48 h, cells were then treated with bortezomib for 24 h, and MTT assay was performed to analyze cell growth. **c** MTT assay and Annexin V/Propidium Iodide (PI) flow cytometry assay of LP1 PA28α knockdown stable cells under exposure to different concentrations of bortezomib or carfilzomib for 48 h. Western blot was performed to show PA28α expression. *P < 0.05, **P < 0.01, Student’s *t* test.

Since PA28α can stimulate 20S proteasome activity both alone and in combination with the PA28β subunit as an 11S complex^25^, we initially anticipated that silencing of PA28α would enhance MM cells' sensitivity to PIs via a reduction in proteasome capacity. We transiently transfected LP1 cell line and primary cells from two MM patients with pooled PA28α siRNA and compared cell growth following exposure to bortezomib. Unexpectedly, silencing of PA28α protected cells from bortezomib-induced cell growth inhibition (Fig. 2a and Supplementary Fig. 1). We further confirmed the regulation of sensitivity to bortezomib through transient overexpression of PA28α in LP1 cells (Fig. 2b). To better characterize the function of PA28α, we generated LP1 and RPMI8226 PA28α knockdown stable cell lines. Consistent with transient transfection, stable knockdown of PA28α blocked bortezomib and carfilzomib-induced cell growth inhibition and apoptosis (Fig. 2c and Supplementary Fig. 2).

### Silencing of PA28α inhibits proteasome activity

**Fig. 3:**
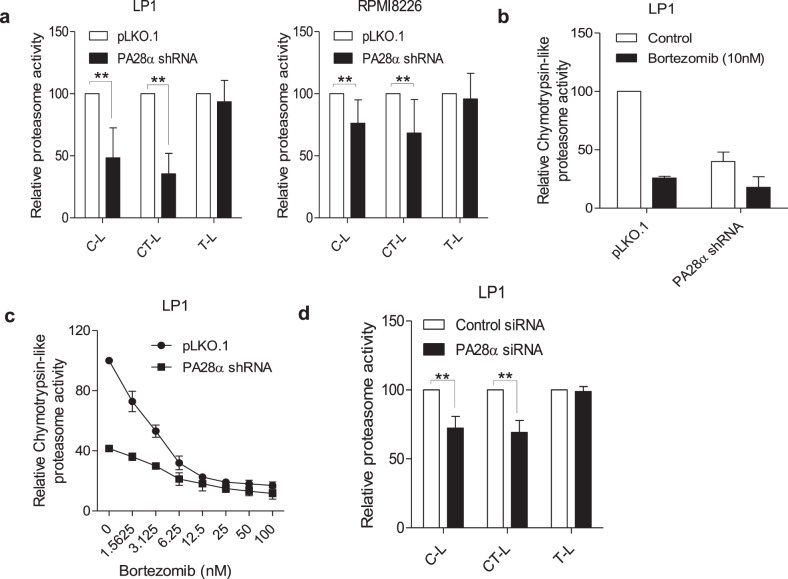
Downregulation of PA28α alters proteasome activity. **a** Proteasome fluorogenic peptidase assays of cell lysates from LP1 and RPMI8226 PA28α knockdown and scrambled control stable cells. **b** LP1 PA28α knockdown stable cells were treated with 10 nM bortezomib for 2 h, cell lysates were obtained, and proteasome fluorogenic peptidase assay was performed to check the chymotrypsin-like proteasome activity, SUC-LLVY-AMC as substrate. **c** LP1 PA28α knockdown stable cells were treated with serial concentrations of bortezomib, chymotrypsin-like proteasome activity was checked as in **b**. **d** LP1 cells were transiently transfected with PA28α siRNA for 72 h, proteasome fluorogenic peptidase assays were performed as in **a**. **P < 0.01, Student’s *t* test.

Proteasomes possess three different enzymatic activities in the 20S proteasome chamber, caspase-like (C-L), chymotrypsin-like (CT-L), and trypsin-like (T-L). Both bortezomib and carfilzomib directly target the proteasome CT-L enzymatic activity via binding the PSMB5 subunit. To explore mechanisms responsible for reduced PI sensitivity induced by PA28α knockdown, we measured proteasome enzymatic activities from the whole-cell lysates of the stable cell lines. In PA28α knockdown cells, C-L and CT-L peptidase activity of the proteasome was significantly reduced, while there was no change of T-L peptidase activity (Fig. 3a). We also compared the proteasome sensitivity to bortezomib by measuring the CT-L protease activity between LP1 PA28α knockdown and control cells. Two-hour treatment with 10 nM bortezomib caused 74% suppression in CT-L protease activity in control cells, but only 55% in PA28α knockdown cells (Fig. 3b). We further observed that under the exposure with escalating concentrations of bortezomib, LP1 PA28α knockdown cells demonstrated less susceptible to bortezomib (Fig. 3c). Transient knockdown of PA28α using pooled siRNA in LP1 cells also demonstrated suppressed C-L and CT-L proteasome activities (Fig. 3d). These data suggest that silencing of PA28α regulated proteasome activity which could manipulate myeloma cells' response to PIs.

### Silencing of PA28α modulates proteasome subunit expression

**Fig. 4:**
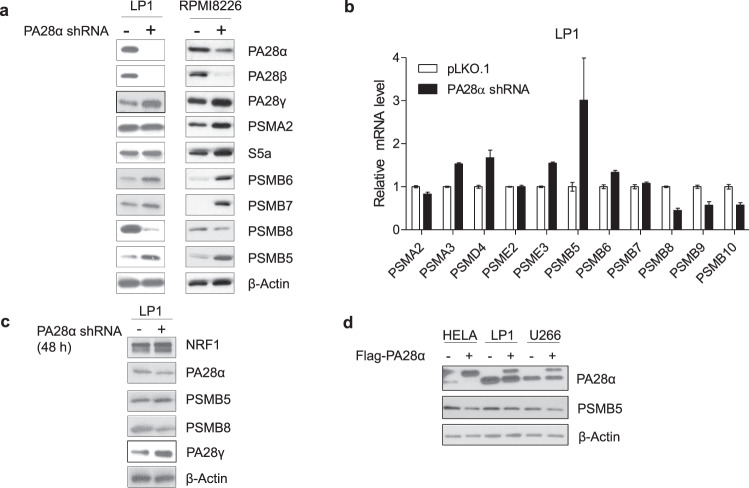
PA28α regulates proteasome subunits expression. **a** Western blot of proteasome subunit expression from LP1 and RPMI8226 PA28α knockdown and control stable cells, β-actin as a loading control. **b** Real-time qPCR assay of proteasome subunits transcript levels from LP1 PA28α knockdown and control stable cells, β-actin as an endogenous control. **c** LP1 cells were transiently transfected with shRNA targeting PA28α or control vector, 48 h after transfection proteasome components expression were checked using western blot. **d** HeLa, LP1, and U266 cells were transiently transfected with Flag-PA28α vector or Flag empty vector, 48 h after transfection, cell lysates were collected, and western blot was performed to check PA28α and PSMB5 expression, β-actin as a loading control.

To further dissect the underlying mechanism of alteration in proteasome activity by PA28α, we first examined proteasome abundancy in PA28α knockdown cells. Although proteasome enzymatic activity decreased in PA28α knockdown cells, expression of constitutive proteasome subunits bearing enzymatic activities such as PSMB5, PSMB6, PSMB7 were increased in PA28α silenced cells, while at the same time, the immunoproteasome subunit PSMB8 was decreased (Fig. 4a). Quantitative real-time PCR confirmed that constitutive proteasome subunits increased in LP1 PA28α knockdown cells, while immunoproteasome subunits decreased (Fig. 4b). We also found increased transcript levels of other proteasome subunits, such as PSMA3, PSMD4 (S5a), PSME3 but decreased expression of PSMB9 and PSMB10 (Fig. 4b). Although the transcript level of PA28β was not changed in PA28α knockdown cells, the protein level decreased significantly. This is consistent with reports from other groups showing that PA28α interacts with and stabilizes PA28β^27^. Transient knockdown of PA28α in LP1 cells also upregulated PSMB5 and PA28γ, with the downregulation of PSMB8 (Fig. 4c). To confirm the regulation of PSMB5 by PA28α, we transiently transfected HeLa, LP1, and U266 cells with a plasmid expressing Flag-PA28α. Consistently, overexpression of PA28α indeed downregulated PSMB5 (Fig. 4d).

### NRF1 is important for PA28α knockdown-induced proteasome regulation

**Fig. 5:**
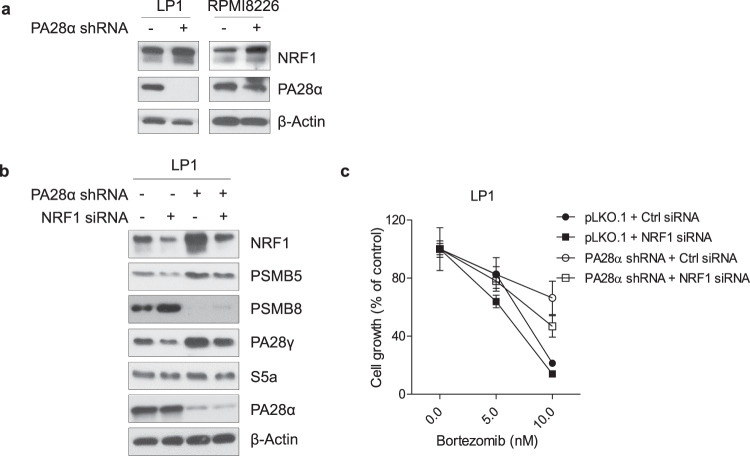
Silencing NRF1 reverses PA28α knockdown-induced proteasome recovery and resensitizes to bortezomib treatment. **a** Western blot analysis of NRF1 in LP1 and RPMI8226 PA28α knockdown and control stable cells. **b** LP1 PA28α knockdown and control stable cells were transiently transfected with NRF1 siRNA or control siRNA. 48 h after transfection, cell lysates were collected and analyzed for expression of proteasome subunits by western blot (**b**) or cells were treated with different concentrations of bortezomib for 24 h, MTT assay was performed to assess relative cell growth (**c**).

In mammalian cells, short-term treatment with PIs induces transcriptional upregulation of proteasome synthesis mediated by nuclear factor erythroid 2-related factor 1 (NRF1 encoded by *NFE2L1* also known as *TCF11*)-dependent proteasome recovery pathway (or proteasome bounce-back)^28^. In our model, we also observed increased expression of NRF1, suggesting the feedback could result from NRF1 activation (Figs. 4c and 5a). To verify the function of NRF1 in PA28α knockdown-induced proteasome regulation, we transiently transfected LP1 PA28α knockdown and control cells with NRF1 siRNA. Knockdown of NRF1 downregulated PSMB5 and PA28γ (Fig. 5b), and also enhanced cell sensitivity to bortezomib (Fig. 5c). The above data suggest that NRF1 is involved in PA28α knockdown-induced proteasome subunits regulation.

### Silencing of PA28α modifies proteasome complex formation

**Fig. 6:**
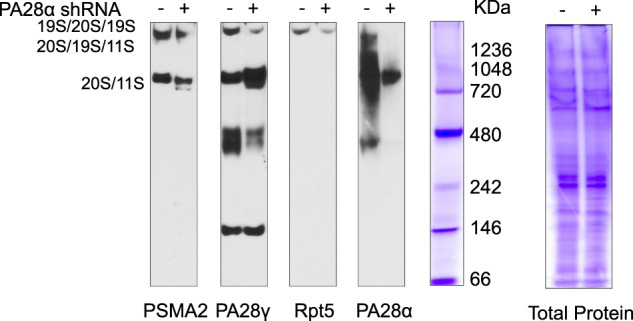
Downregulation of PA28α changes proteasome complex formation. LP1 PA28α knockdown and control stable cells were lysed in proteasome lysis buffer, same amounts of cell lysates were loaded on native PAGE, and proteasome complex was checked followed by western blot. Total protein was also visualized by Coomassie Blue R-250 staining.

The discrepancy between proteasome abundance and proteasome activity prompted us to explore how silencing of PA28α altered proteasome function. We examined proteasome formation by checking the proteasome pool using native PAGE from cell lysates of LP1 PA28α knockdown stable cell lines. In PA28α knockdown cells, the formation of 19S/20S/19S double-capped proteasome decreased, with the increased formation of PA28γ capped proteasomes (Fig. 6). Among the three 11S proteasome subunits, α and β can activate all three types of protease activities, while γ can only activate T-L protease activity^29^, the increased formation of PA28γ-bearing proteasomes explained our previous observation that there was a significant decrease in C-L and CT-L protease activities in knockdown cells but no difference on T-L protease activity.

### Silencing of PA28α decreases proteasome load

**Fig. 7:**
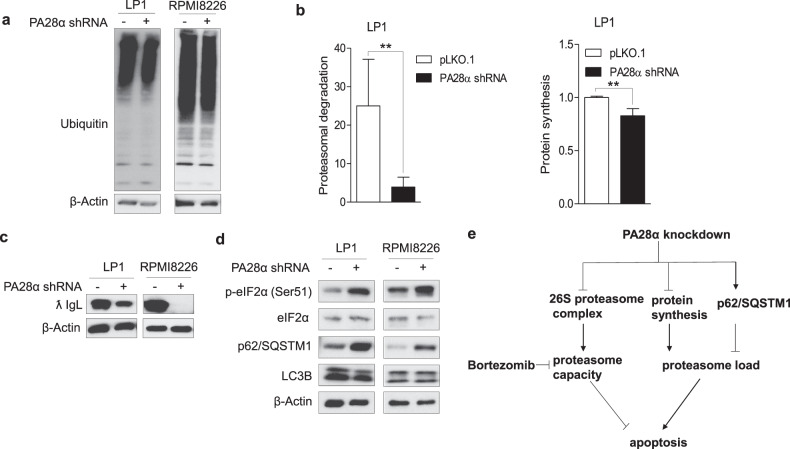
Downregulation of PA28α decreases proteasome workload. **a** Western blot analysis of ubiquitinated proteins in total protein lysates extracted from LP1 and RPMI8226 PA28α knockdown stable cells. β-actin as a loading control. **b** OPP pulse-chase assay of proteasome degradation and protein synthesis in LP1 PA28α knockdown stable cells. Western blot analysis of immunoglobulin lambda light chain (ƛ IgL) (**c**), eIF2α, p62/SQSTM1, and LC3B (**d**) in LP1 and RPMI8226 PA28α knockdown stable cells, β-actin as a loading control. **P < 0.01, Student’s *t* test. **e** Working model of PA28α knockdown in MM.

Since PA28α knockdown inhibited proteasome activity, we expected that impaired proteasome activity could induce proteasome stress with the accumulation of protein–ubiquitin conjugates in the cell. Although proteasome activity is suppressed in PA28α knockdown cells, we did not detect more accumulated ubiquitinated proteins (Fig. 7a). In MM cells, sensitivity to PIs is closely correlated with proteasome load versus capacity. Cells with higher proteasome load and lower proteasome capacity show more sensitivity to PIs^30,31^. We asked whether knockdown of PA28α could also decrease proteasome load within the cells. We performed a pulse-chase assay to compare the proteasomal degradation. Cells were labeled with O-propargyl-puromycin (OPP) for 30 min and chased for 2 h with or without 40 nM bortezomib after thoroughly washing. The ratio of labeled protein degradation through proteasome was significantly lower in PA28α knockdown cells (Fig. 7b). In multiple myeloma cells, the proteasomal load is contributed mainly by the extensive synthesis of immunoglobulin^31,32^. We also found that in PA28α knockdown cells, the expression of the immunoglobulin light chain was greatly inhibited (Fig. 7c). Reduction in proteasome load is quite interesting and requires further investigation. Based on our observation that PA28α knockdown activated eIF2α (Fig. 7d), the regulation could result from protein synthesis inhibition in PA28α knockdown cells. Importantly, OPP-labeling also demonstrated that nascent protein synthesis was suppressed in PA28α knockdown cells (Fig. 7b). We also observed that although silencing of PA28α did not induce autophagy, which is active in these cells, however, it did upregulate p62/SQSTM1 protein levels (Fig. 7d) which could protect cells under proteasome deficiency by enhancing the delivery of ubiquitinated proteins to the autophagy pathway. The above data demonstrated that knockdown of PA28α reduced both cellular proteasome activity and proteasome load, resulting in new protein homeostasis with less dependent on proteasome for protein degradation and subsequent PI resistance (Fig. 7e).

## Discussion

Proteasome inhibitors are a standard part of front-line therapy in MM, and have continued to improve the outcomes for MM patients. Unfortunately, the intrinsic and acquired resistance to PIs limits their long-term benefit in the clinic. Many factors have been identified that contribute to PI resistance, including proteasome gene mutations and upregulation, alterations of protein expression involved in cell stress response, survival, and apoptotic pathways^4–7^, but many of the key underlying mechanisms remain unclear.

The proteasome load versus capacity ratio influences cell sensitivity to PIs, which has been verified both in intrinsic and acquired PI resistance. Increasing proteasome load or decreasing proteasome capacity could disturb the balance and result in PI sensitivity. Researchers have been focused on modulating the proteasome capacity portion of the equation by targeting specific proteasome subunits. Unfortunately, targeting the expression of different proteasome subunits results in distinct cell responses to PIs. Evidence from human cancer cell lines has indicated that silencing of the 20S core particle subunits reduced cell viability upon PI exposure. In contrast, downregulation of the 19S proteasome subunits protects cells from treatment with PIs. The mechanism of 19S reduction-induced PI resistance involves reduced proteotoxic stress and a broad change of proteasome substrates mediating cell survival and proliferation^33,34^. NRF1-dependent induction of proteasome subunit expression and subsequent rescue of proteasome function in response to PIs has been well studied. 19S subunit reduction does not activate NRF1 and the expression of 20S subunits is unchanged^34^. In our present study, we demonstrated that knockdown of the 11S proteasome activator, PA28α, activated NRF1-mediated proteasome recovery response. Moreover, although some of the proteasome subunits were upregulated in PA28α knockdown cells, the composition of the 26S proteasome complex was reduced, with an accordingly increased assembly of PA28γ capped proteasome. The reason why cells adapt to PA28α knockdown via assembly PA28γ incorporated proteasome is still unknown and warrants further investigation. Rapid formation of PA28 or PA200 bearing alternative proteasome has also been observed in rabbit reticulocytes lysate (RRL) and human lung fibroblasts under exposure to bortezomib^35,36^. Furthermore, alterations in 20S composition such as the α4–α4 proteasome, mediates cell resistance to toxic metal ions in human cells under the regulation of multiple oncogenic signals^37^. The underlying mechanism and function of such alternative proteasomes remains to be further determined but suggests an essential role in helping cells respond to environmental stresses.

In addition to the decreased proteasome activity, knockdown of PA28α also downregulated proteasome load which may result from the inhibition of protein synthesis and upregulation of p62SQSTM1. How cells responded to proteasome inhibition by lowered protein synthesis is quite important and under further investigation. In myeloma, excessive immunoglobulin production and accumulation cause severe complications in patients, eradicating the overproduction of immunoglobulin could provide a promising therapeutic strategy. Altogether, although knockdown of PA28α inhibits proteasome activity, proteasome load within the cells decreased concurrently, resulting in the new protein homeostasis.

Until now, the function of PA28α/β remains partly described. Some groups have reported PA28α/β participates in MHC class I antigen presentation, but the underlying mechanism is controversial and not clear^20,38–41^. Others have suggested that PA28α/β may play roles in protein degradation and antioxidant response^27^. Here, based on our observations of PA28α knockdown cell lines, the function of PA28α comprises at least two key aspects in MM cells. On the one hand, PA28α can regulate cell growth and proliferation. Expression of PA28α is upregulated in MM cells comparing to normal plasma cells as well as during disease progression in vivo. Silencing of PA28α significantly suppresses MM cell proliferation (Supplementary Fig. 3), suggesting that PA28α is important for myeloma progression. On the other hand, PA28α can regulate proteasome biogenesis and activity directly or indirectly. Silencing of PA28α reduced proteasome load and proteasome stress within the cell, sustained protein homeostasis with a lower level of proteasome activity leading to PI resistance. In our observation, the expression level of PA28α may not be correlated with intrinsic cell sensitivity to bortezomib. As we compared MM cell lines under the exposure to bortezomib, sensitivity to bortezomib was not associated with the basal level of PA28α (Supplementary Fig. 4). While manipulating the PA28α protein level could impact cell response to PIs as evidenced by data from transient knockdown or overexpression of PA28α within MM cells. Altogether, in this report, we describe the function of PA28α in MM cells, also provide novel insights into regulating PIs sensitivity through modulation of the 11S proteasome subunit PA28α.

## Materials and methods

### Reagents, constructs, and antibodies

Bortezomib and carfilzomib were purchased from selleck chemicals. Other reagents were purchased from puromycin (Sigma), cycloheximide (Sigma), SUC-LLVY-AMC (Enzo Life Science), Z-Leu-Leu-Glu-AMC (Enzo Life Science), Boc-Leu-Arg-Arg-AMC (Enzo Life Science). Antibodies used were as follows: PA28α (Cell Signaling), PSMA2 (Cell Signaling), S5a (Cell signaling), PA28β (Cell Signaling), Phospho-eIF2α (Ser51) (Cell signaling), eIF2α (Cell signaling), α-tubulin (Genetex), PA28γ (Genetex), PSMB5 (Genetex), PSMB6 (Enzo life science), PSMB7 (Genetex), PSMB8 (Genetex), PSMB9 (R&D systems), PSMB10 (R&D systems), Rpt5 (Enzo life science), Ubiquitin (Cell Signaling), β-actin (Santa Cruz Technology), TCF11/NRF1 (Cell Signaling), LC3B (Cell Signaling), p62/SQSTM1 (MBL International) human Ig lambda light chain (R&D systems), actin (Sigma). pLKO.1 empty vector, shRNA vector targeting human PA28α, NRF1 siRNA (ON-TARGETplus SMARTpool), PA28α siRNA (Accell SMARTpool), and control siRNA were purchased from Dharmacon. Native Mark unstained protein standard was purchased from life technologies.

### Generation of PA28α knockdown stable cell lines

To generate PA28α knockdown stable cell lines, LP1 and RPMI8226 cells were infected with recombinant lentiviruses carrying PA28α knockdown shRNA generated in 293T cells. Sequences for PA28α knockdown were: TAACACAGCATAAGCATTGCG (shRNA #1), TTTCATTGCAGTTCACTGGGC (shRNA #2). Infected cells were then selected in a medium with 1.25 ng/ml puromycin. The puromycin-resistant cells were expanded and tested with PA28α antibody before experiments.

### Cell growth assay

Cell growth was assessed using Cell Proliferation Assay kits from atcc.org following the manufacturer’s procedure.

### Cell apoptosis assay

Cell apoptosis was analyzed using FITC-annexin V/PI staining (BD Biosciences) following the manufacturer’s protocol. Data were collected and analyzed using FlowJo (Treestar, Ashland, OR) software.

### Western blot

Western blot was performed as described previously^42^.

### Proteasome fluorogenic peptidase assays

Proteasome fluorogenic peptidase assays were performed on whole cell lysate. In brief, cells were collected and lysed in proteasome lysis buffer (50 mM Tris-HCl (pH 7.5), 5 mM MgCl_2_, 0.5 mM EDTA, 10% glycerol, 0.25% NP-40,1 mM DTT, 2 mM ATP). The extracts were cleared by centrifugation at 16,000×*g* for 30 min at 4 °C. The same amount of proteins were transferred to a 96-well microtiter plate (BD Falcon). To measure the proteasome peptidase activity, three different kinds of substrates were used, caspase-like: Z-Leu-Leu-Glu-AMC, chymotrypsin-like: Suc-Leu-Leu-Val-Tyr-AMC, trypsin-like: Boc-Leu-Arg-Arg-AMC. The substrate was added to the plate to a final concentration of 50 µM and mixed, incubated at 37 °C for 30 min. Fluorescence was monitored on a microplate fluorometer SpectraMAX (Molecular Devices) using wavelength 380-nm excitation, 460-nm emission.

### Real-time qPCR

Real-time qPCR was performed as described previously^42^. Primers used were KiCqStart SYBR Green Primers obtained from Sigma.

### Native gel electrophoretic mobility assays

Native gel electrophoresis was performed, as described previously^8^. Cell extracts and Native Mark unstained protein standard were also resolved in the gel and followed by Coomassie Blue R250 staining.

### Proteasomal-mediated degradation assay

Proteasomal-mediated degradation was evaluated using a protein synthesis assay kit from Cayman chemical according to the manufacturer’s manual with a small modifications. Briefly, cells were labeled using O-propargyl-puromycin (OPP) for 30 min then washed and incubated with complete media with or without 40 nM bortezomib for 2 h. After the incubation, cells were fixed and stained with 5-FAM azide for 30 min. After washing, cells were collected and analyzed using flow cytometry. Proteasomal-mediated degradation was calculated using the percentage of bortezomib inhibited peptides degradation relative to the peptides at the end of OPP labeling.

### Patient samples

Bone marrow aspirates from MM patients were prepared following Emory University Institutional Review Board approved consent. Buffy coat cells were collected as described previously^42^ and applied in experiments.

### Analysis of plasma cell and myeloma gene expression

Affymetrix microarray data were downloaded from GEO data sets GSE6477 and log2 transformed. Data were plotted in R/Bioconductor^43^ using the function “boxplot”. Statistical significance between groups was determined relative to plasma cells from healthy donors using linear regression where disease stage (e.g., MGUS, SMM, NDMM, RRMM) was treated as a factor.

## Supplementary information

BCJ checklist

Supplementary figures
