# Supplementary material for: Downregulation of PA28α induces proteasome remodeling and results in resistance to proteasome inhibitors in multiple myeloma
Source: Blood Cancer J. 2020 Dec 14;10(12):125. doi: 10.1038/s41408-020-00393-0 (PMC7736847; doi:10.1038/s41408-020-00393-0)
Supplement: Supplementary file 2 — Supplementary figures [file 41408_2020_393_MOESM2_ESM.pdf]

Supplementary Figures

Figure 1

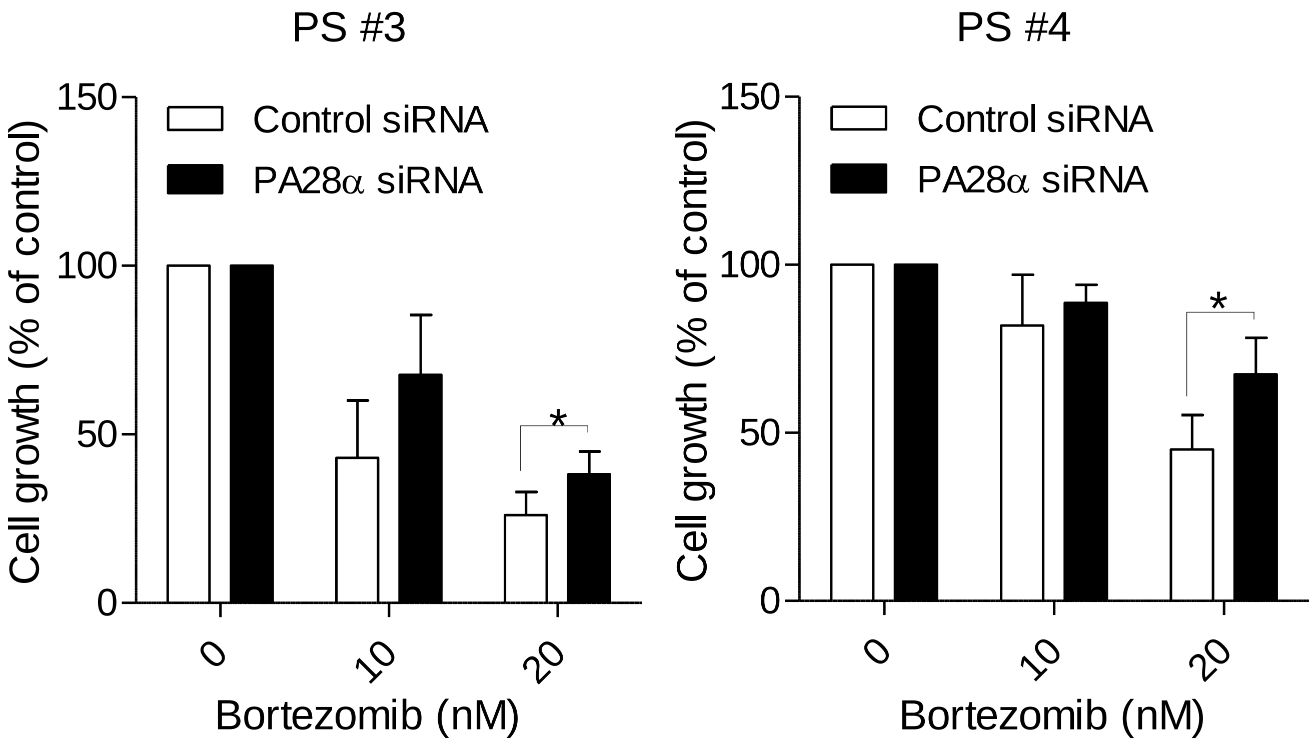

Fig. 1 PA28α regulates multiple myeloma sensitivity to PIs. The primary cells from patients #3 and #4 were transiently transfected with PA28α siRNA or control siRNA. 72 hours after transfection, cells were resuspended in fresh media and treated with 10 or 20nM bortezomib for 24 hours, cell growth assay was performed using MTT assay. * P<0.05, Student's t-test.

Figure 2

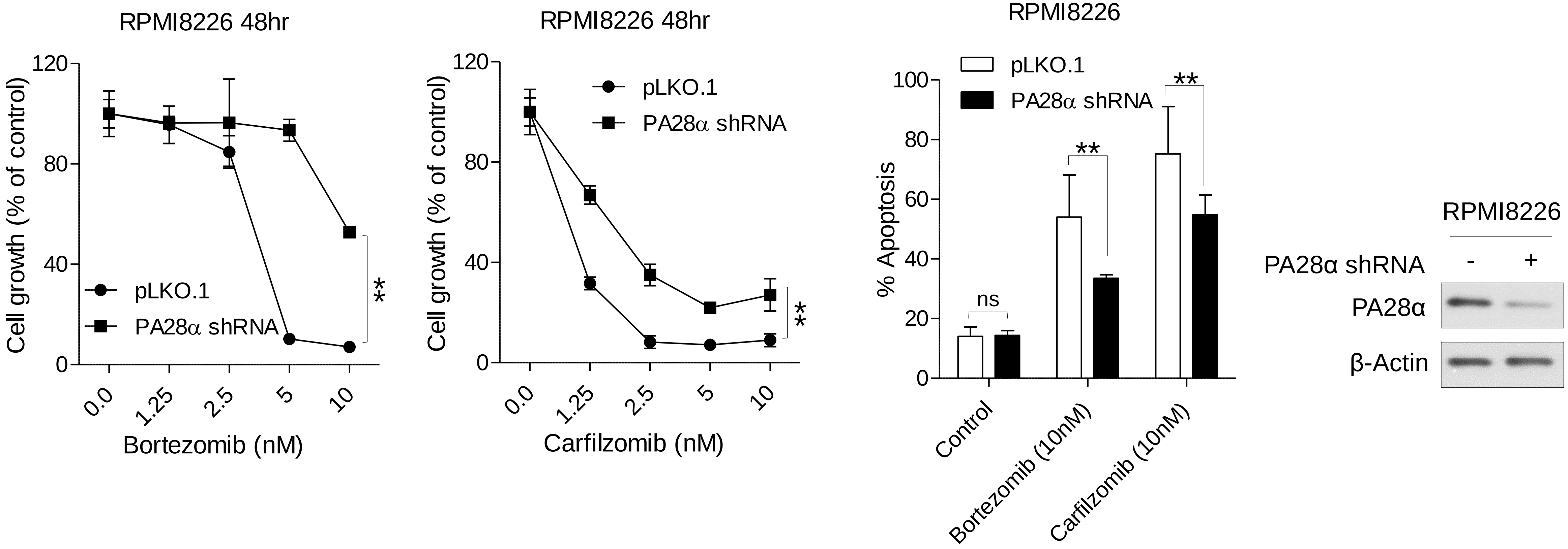

Fig. 2 PA28α regulates multiple myeloma sensitivity to PIs. MTT assay and Annexin V/Propidium Iodide (PI) flow cytometry assay of RPMI8226 PA28α knockdown stable cells under exposure to different concentrations of bortezomib or carfilzomib for 48 h. Western blot was performed to show PA28α expression. ** $P<0.01$, Student's t-test.

Figure 3

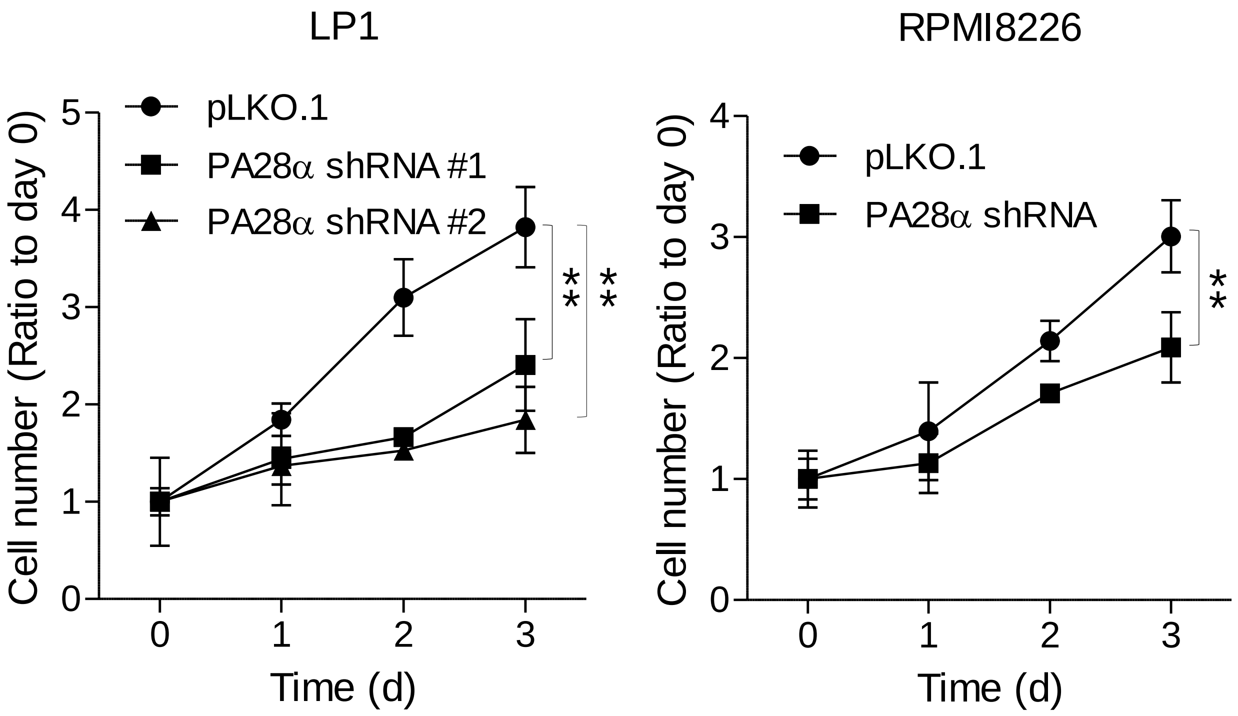

Fig. 3 PA28α knockdown inhibits cell proliferation. Cell proliferation assay of LP1 and RPMI8226 PA28α knockdown and control stable cells. ** $P<0.01$, Student's t-test.

Figure 4

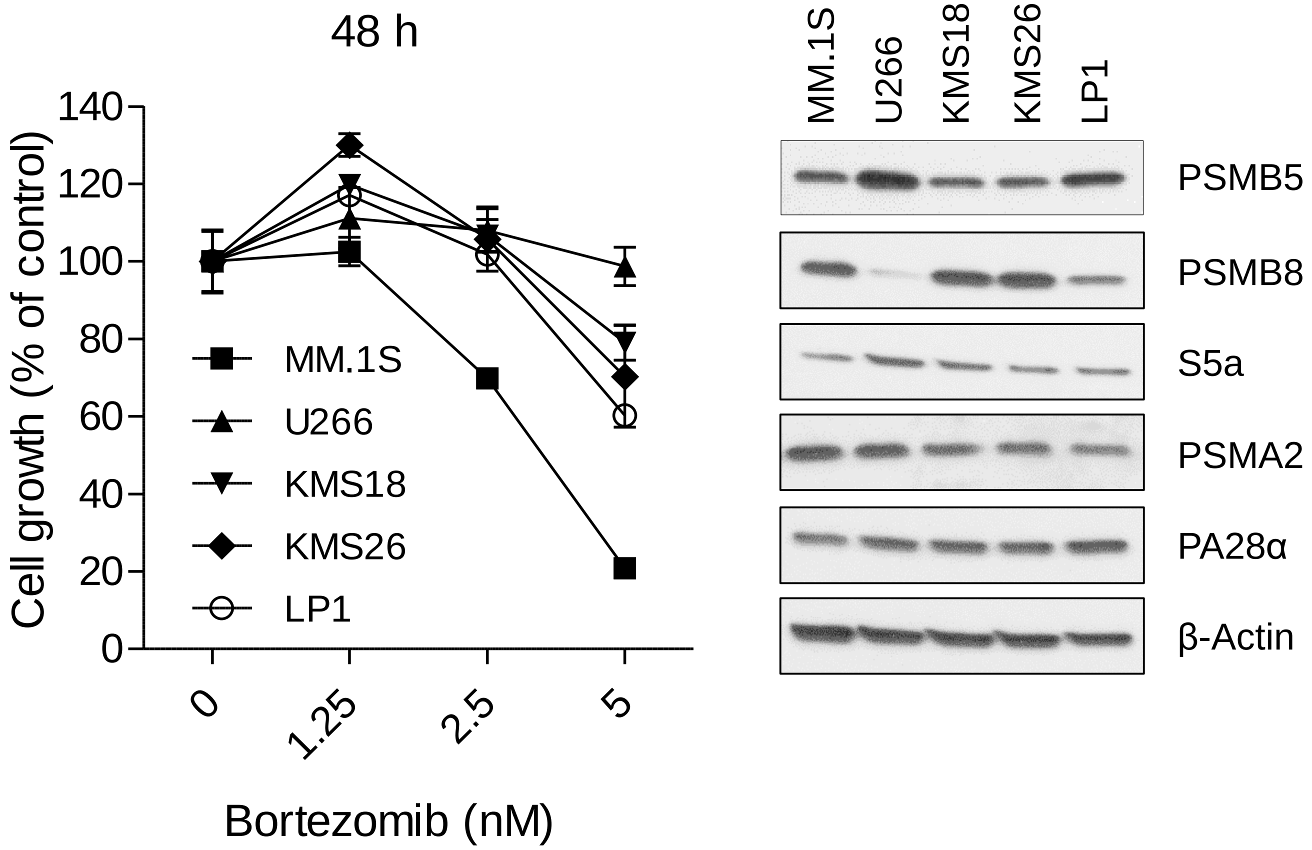

Fig. 4 MM cell lines proteasome subunits expression and sensitivity to bortezomib. MM cell lines were treated with bortezomib for 48 h and cell growth was assessed using MTT assay. Proteasome subunits expression was analyzed by western blot, β-Actin as loading control.
